# Climate change in healthcare: Exploring the potential role of inhaler prescribing

**DOI:** 10.1002/prp2.675

**Published:** 2020-10-30

**Authors:** Joachim Starup‐Hansen, Henry Dunne, Jonathan Sadler, Anna Jones, Michael Okorie

**Affiliations:** ^1^ Brighton & Sussex Medical School Brighton UK; ^2^ Brighton & Sussex University Hospitals Brighton UK

**Keywords:** climate change, inhalers, sustainable prescribing

## Abstract

Climate change has been described as the biggest global health threat of the 21st century. As a result, governments around the world are committing to legislative change in order to reduce greenhouse gas emissions (GHGEs). The healthcare sector makes a significant contribution to GHGEs and in line with national legislation in the UK, the NHS has recently committed to achieving net zero emissions by 2050. The management of asthma and COPD largely depends on the prescribing of medications that are delivered through inhalers. In the UK, the use of pressurized metered dose inhalers (pMDIs), which rely on hydrofluorocarbon (HFC) propellants accounts for 3.5% of the NHS’s total carbon footprint. In contrast, dry powder inhalers (DPIs) have a much lower carbon footprint due to the absence of a HFC propellant. Here we review evidence of the impact of inhaler choices across four domains: environmental impact, clinical effectiveness, cost effectiveness and patient preferences. We find that as well as a lower global‐warming potential, DPIs have additional benefits over pMDIs in other domains and should be considered first line where clinically appropriate.

## Graphical abstract

A comparison of dry powder inhalers (DPIs) and pressurised metered‐dose inhalers (pMDIs) across clinical, social and environmental domains.

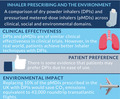

## 1 INTRODUCTION

**Figure 1:**
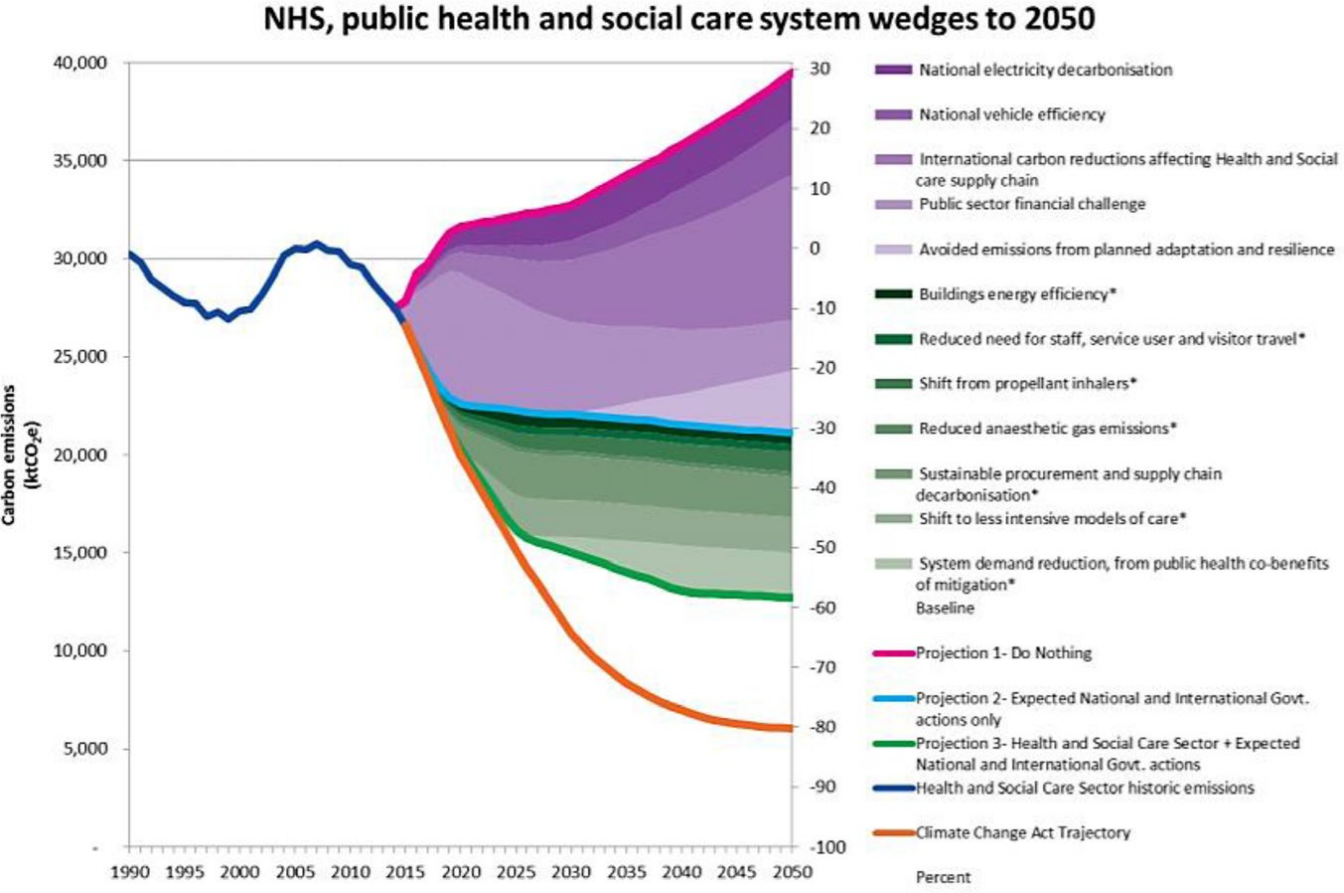
This graph shows the potential impact of a shift from propellant inhalers on carbon emissions in the context of other global, national and health sector actions^14^

Climate change has been described as the “greatest threat to global public health in the 21st century”.^1^, ^2^ On 1 May 2019, the United Kingdom was the first country in the world to declare a “climate emergency”^3^ in response to the Intergovernmental Panel on Climate Change report in 2018. The primary method for combating climate change is to reduce greenhouse gas emissions (GHGEs)^4^ and the UK government has committed to achieving net zero GHGEs compared to a 1990 baseline by 2050.^5^, ^6^ In order to achieve this goal, a number of sectors will need to reduce their emissions. One of these sectors is the UK National Health Service (NHS), which is the UK’s largest public sector greenhouse gas emitter.^6^, ^7^, ^8^ Importantly, around 3.5% of the NHS’s carbon footprint is derived from a single treatment ‐ pressurized metered dose inhalers (pMDIs) ^8^, ^9^, ^10^, ^11^ which are used in the management of asthma and chronic obstructive pulmonary disease (COPD). The inhaler constituent responsible for 96% of pMDI’s global‐warming potential (GWP), is not the active ingredient but rather the propellant.^3^, ^12^ Dry powder inhalers (DPIs) which rely on a propellant‐free mechanism that significantly reduces the environmental impact of the prescription are the favored choice in a number of European countries^13^—although this is also due to nonenvironmental factors such as local manufacturing. A priority of the UK’s Sustainable Development Unit (SDU) strategy is to achieve an 80% reduction in NHS’s GHGE by 2050 and this involves switching from propellant to DPI inhalers which have a lower GWP (Figure 1).^6^, ^14^

Climate change and its related health risks^15^ is a driver for the reduction in the number of pMDIs prescribed in the UK. Here we review the evidence for the environmental impact of pMDIs, which is now well established.^6^, ^16^, ^17^, ^18^ We also consider the clinical and cost effectiveness and patient preference of DPI vs pMDI inhalers and propose that these factors are not necessarily barriers to switching to inhaler devices with lower GWP.

## 2 ENVIRONMENTAL IMPACT

pMDIs have a significantly higher GWP, due to their hydrofluoroalkane (HFA) propellants, which are quoted to be, gram for gram, up to 3800 times a more powerful greenhouse gas than carbon dioxide.^10^, ^19^ The DPI, which does not require a propellant, has a carbon footprint 18 times lower than MDIs.^18^, ^19^, ^20^ Wilkinson et al^17^ calculated that if 10% of UK pMDI inhalers were replaced with their corresponding DPI devices, more than 68.6 kilo tonnes of CO_2_ equivalent could be saved each year. This is equivalent to the mass of CO_2_ produced from 43 thousand roundtrip transatlantic flights (return economy flights from London to New York).^21^

A study comparing carbon footprints of the entire life‐cycle of various inhalers including their productive and distribution found that MDI devices had up to 30 times larger carbon footprints than DPI equivalents and that these differences were mainly related to the use phase and end‐of‐life (disposal) phase.^22^ It was calculated that if England applied the Swedish distribution of pMDIs and DPIs, 550 kt of CO_2_ would be saved annually—which corresponds to approximately 2.6% of NHS England’s total carbon footprint.^22^

Furthermore, the inappropriate disposal of pMDIs contributes further to their environmental impact.^23^ Although some pMDIs do have dose counter mechanisms, this is not universal (in contrast to DPIs) and means that patients may not know how many doses are left in their inhalers. This creates two problems: firstly, patients can unknowingly run out of doses which is potentially catastrophic during an acute exacerbation of asthma or COPD; and secondly, it leads to patients being more likely to request repeat prescriptions earlier than necessary.^24^ The inappropriate disposal of pMDI devices with unused doses is especially concerning as not only does it increase the prescribing burden but devices no longer in use continue to release greenhouse gases into the atmosphere.^23^ In a UK study, inhalers that had been disposed of incorrectly were collected over 90 days from one local district hospital and this resulted in enough pMDIs to produce an equivalent of 2.63 tonnes of CO_2_ emissions which would otherwise have been released into the atmosphere.^23^ The recent British Thoracic society position statement on “The Environment and Lung Health”^25^ highlights the importance of advising patients on avoiding disposal of inhalers in landfill sites and of supporting recycling schemes through pharmacies.

It should be noted that DPIs have limited benefit in some environmental domains not related to climate change. This is seen in Jeswani et al’s study^26^ which found that although DPIs can reduce the climate change impact of inhalers by 96% if they replace pMDIs, this comes at a cost in domains such as marine eutrophication and fossil depletion, which should be acknowledged.

In view of the legal obligations to reduce GHG emissions together with the health benefits of climate change mitigation, a move away from pMDIs to DPIs seems increasingly important.

## 3 CLINICAL EFFECTIVENESS

Concerns about the effectiveness of each inhaler needs to be addressed. In 2005, Dolovich et al’s^20^ systematic review found no significant differences in the efficacy of different inhaler types, including pMDIs and DPIs in both asthma and COPD patients in all clinical situations examined. However, the reviewed RCTs only included patients with a perfect inhaler technique. This is important, as the conclusion may not represent the “real world efficacy”, in which patients do not always use their inhalers correctly.

pMDIs deliver the drugs into the airways through a propellant mechanism. Failure in coordinating actuation of the inhaler with a long slow inspiration by the patient is the most significant problem for patients using pMDIs.^27^ When using DPIs, the drug is pulled into the lungs as a result of the patients’ own inspiratory effort and therefore, when using a DPI, poor coordination becomes less of a barrier to effective therapy.^28^ In fact, it has been widely reported that the proportion of patients using the correct technique with pMDIs without a spacer is low. Two separate studies reported correct technique using a pMDI as compared with DPIs to be 23%‐43% and 53%‐59% respectively.^18^, ^22^ Effective use of a DPI requires the patient to generate a peak inspiratory flow (PIF) that overcomes the internal resistance of the inhaler. Using a Turbohaler^©^, PIFs >30 L/min have been shown to be sufficient for effective administration in both adult and children populations.^29^, ^30^ As disorders primarily affecting expiration, both COPD and asthma do not significantly limit individual PIF rates. However, clinicians should be aware that, DPIs are not the appropriate choice of inhaler for patients who are not able to generate sufficient inspiratory flow. For example, frail, elderly patients, selected patients with COPD, very young patients or those with muscle weakness. Pederson et al^29^ showed that 25% of children 3‐5 years of age were not able to, and Janssens et al^31^ showed that up to 30% of elderly COPD patients were not able to achieve rates of 45 L/min via various DPI devices.

Nonetheless, when considering effectiveness of drug delivery, DPIs should be considered in clinical scenarios where both inhaler types are appropriate while recognizing that some patients will need to be provided with a pMDI in a rescue pack for use in the case of an acute exacerbation. The UK National Institute for Health and Care Excellence (NICE) has produced a patient decision aid for patients with asthma that suggests a DPI device is suitable and should be considered for asthma patients who can breathe in deeply for 2‐3 seconds independent of their coordination or ability to take a longer 4‐5 second breath.^32^

## 4 COST EFFECTIVENESS

The apparent higher financial cost associated with DPIs compared to pMDIs has long been a barrier to their widespread use in the NHS.^17^, ^27^ This is because the pMDI version of the frequently prescribed short‐acting bronchodilators for asthma and COPD are less expensive than their DPI counterparts.^17^, ^33^ However, other medications, especially combination inhalers of Long‐Acting‐Beta‐Agonists and Inhaled Corticosteroids are cheaper in DPI formulations compared to pMDIs.^17^ Interestingly, a cost analysis of community inhaler prescriptions concluded that although a widespread switch from pMDI to DPIs would result in an increase in short‐acting bronchodilator associated costs, this would be offset by the savings made with other inhaled medications. Overall, it was modeled that for every 10% of pMDIs that are replaced by cheapest DPI alternatives, more than £8.24 million could be saved annually.^17^ Similarly, Korean and South Indian studies have failed to show a significant difference in overall costs between DPI and pMDI.^34^, ^35^, ^36^ This renders the historical assumption of DPIs being more expensive than pMDIs as questionable and hence should not be seen as a barrier to DPI prescriptions. In fact, the modeled economic benefits of switching to DPIs provides a further driver to change inhaler devices prescribing practice.

## 5 PATIENT PREFERENCE

Unsurprisingly, ease of use has been identified as one of the most important considerations for patients and healthcare professionals in the choice of inhalers.^37^ The 2017 study by Ramadan et al^38^ explored the satisfaction of 246 asthma and COPD patients treated with either DPI and/or pMDI and discovered that a significantly greater percentage (73% vs 46%) of DPI users found their inhaler easy to use and were more satisfied with their care compared to pMDI users. Furthermore, a 2011 study^39^ investigated the response when COPD patients previously treated with pMDI were switched to a DPI and given inhaler training during a hospital visit for an acute exacerbation of their condition. Findings indicate that 3 months after the switching intervention, the majority of switched patients remained on the DPI. This not only illustrated that some patients may have preferred using a DPI but also that it is feasible to make simple interventions to switch patients currently treated with a pMDI to a DPI successfully. It is important to note that nonconsensual switching of inhalers, without appropriate instruction regarding inhaler techniques can lead to worse clinical outcomes for COPD and asthma patients.^40^ However, Price et al^41^ have shown that outcomes after consensual switching of inhalers with face‐to‐face online consultations did not lead to any reduction in clinical effectiveness. In addition, a more recent study^42^ attempted to address the paucity of information about patients and physician attitudes to the choice of inhalers and surveyed 50 NHS patients who were already using their inhalers. This found that 44% of those patients expressed that the carbon footprint of their inhaler is “important” or “very important” to them. We concede that this is a small sample size and that more research into this area is required. Importantly, in line with other research, it also found that 80% of those surveyed rated ease of use of inhalers as “important” or “very important”. Overall, using a patient‐centered approach to inhaler prescribing that takes into account patients’ preferences is important. Some patients will choose to use or remain on a pMDI but there is evidence that for some patients a DPI will be preferable. Device options should be discussed with patients and clinicians should feel confident to start a DPI or switch a pMDI inhaler to a DPI when clinically appropriate if patients are in agreement. Appropriate advice as well as regular technique reviews should also be offered.

## 6 CURRENT GUIDELINES

Currently, guidelines in the UK regarding the choice of inhalers do not make an appropriate emphasis on the potential carbon savings that could be achieved when selecting DPIs over pMDIs. In fact, the July 2019 NICE guidance for COPD diagnosis^43^ and management does not offer any advice regarding deciding between pMDIs and DPIs and contains no information on the environmental impact of each device. However, in asthma management, the recent British Thoracic Society (BTS) guidance does now advise that “Pr*escribers, pharmacists and patients should be aware that there are significant differences in the global‐warming potential of different MDIs and that inhalers with low global‐warming potential should be used when they are likely to be equally effective”*.^34^ This is an encouraging development but is short of explicitly recommending the DPIs (which have a ‘lower global‐warming potential’) as the preferred choice based on similar efficacy, higher patient preference and lower climate impact. The patient decision aid on inhaler devices for asthma published by NICE also includes a discussion of the environmental impact of different inhalers.^32^

Although there is a lack of research into clinician awareness of the carbon footprint of different inhaler types, there is certainly scope to increase this awareness and potentially alter prescribing decisions through a greater emphasis in clinical guidelines. For the busy clinician, guidelines should highlight boldly that DPIs are the preferred inhaler type under circumstances when both pMDIs and DPIs are appropriate for the patient.

## 7 CONCLUSIONS AND RECOMMENDATIONS

Climate change is an existential threat to the entire population of this planet and because of this, all societal sectors must adopt changes to reduce GHG emissions. A shift in inhaler devices prescribing practices in favor of DPIs and away from pMDIs, where clinically appropriate, has the potential to significantly reduce healthcare associated GHGEs.^6^ Moreover, as well as the well‐documented environmental benefits, a switch towards DPI devices should be supported as DPIs are at least as effective, potentially cheaper and are often are preferred by patients.

In accordance with the NHS Long Term Plan to “shift to lower carbon inhalers”^44^ our recommendations are that national and local guidelines are updated; guidance should consider the potential benefits of advising DPIs as the device of choice in new diagnoses of asthma and COPD as well as the benefits of switching patients currently using pMDIs to DPIs where clinically appropriate. We recognize the short‐term financial impact on the local health economy but also highlight potential long‐term cost savings.

Some concerns have been expressed in response to the proposals to reduce pMDI prescribing in the UK.^45^ In line with others,^8^ we are not advocating for a total switch from pMDIs to DPIs and recognize that some patients will have valid reasons to continue to use a pMDI but instead aim to explore the relative advantages and disadvantages of changing prescribing practice. It is the role of the healthcare professional to ensure that their patient’s condition is managed optimally with appropriate attention paid to their preferences and that patients are able to manage their symptoms effectively in partnership with their clinical team.

We recognize that HFCs are widely used globally across many different sectors such as air conditioning and refrigeration and their use in inhalers represents only a small proportion of this volume; the counter‐argument to this is that action is being taken to reduce this across these sectors^46^ and healthcare should not be exempt from being required to take action. Equally, while pMDIs contribute only a small proportion of the carbon footprint of the NHS, if this can be reduced with no negative impacts on patients then we would argue that this should be prioritized along with other measures taken to make healthcare more sustainable. It is therefore appropriate that this is also addressed within the healthcare sector and that healthcare workers, as professionals advocating for the health of the public that they serve, would want to ensure that the contribution of healthcare to climate change is minimized as far as possible.

Future research should focus on evaluating clinician awareness of the environmental benefits of DPI inhalers and evaluating the impact of a change in guidelines on the prescribing of inhalers. Finally, timely implementation of a large number of environmental measures is essential to reverse the environmental catastrophe that is currently unfolding.

## CONFLICT OF INTEREST

The authors declare no conflict of interest.

## ETHICAL STATEMENT

Ethical and legal approval was not required for this paper.

## Data Availability

Data sharing is not applicable to this article as no new data were created or analyzed in this study.
